# Effect of PAH Specific Therapy on Pulmonary Hemodynamics and Six-Minute Walk Distance in Portopulmonary Hypertension: A Systematic Review and Meta-Analysis

**DOI:** 10.1155/2014/528783

**Published:** 2014-11-16

**Authors:** Muhammad Faisal, Furqan Siddiqi, Ahmad Alkaddour, Abubakr A. Bajwa, Adil Shujaat

**Affiliations:** ^1^Division of Pulmonary, Critical Care and Sleep Medicine, University of Florida, Jacksonville, FL 32209, USA; ^2^Department of Medicine, University of Florida, Jacksonville, FL 32209, USA

## Abstract

*Background*. Little is known about the effect of pulmonary arterial hypertension (PAH) specific therapy on pulmonary hemodynamics and exercise capacity in patients with portopulmonary hypertension (PoPH) because such patients are usually excluded from randomized clinical trials (RCT) of such therapy. *Methods*. We searched PUBMED using the terms “(Therapy/Broad (filter)) AND (portopulmonary hypertension).” We included studies that met the following criteria: ≥5 patients, AND PoPH confirmed by right heart catheterization (RHC), AND follow-up RHC data, AND/OR baseline and follow-up 6MWD available. *Results*. 12 studies met our inclusion criteria. None was a RCT. The baseline mPAP was 48.6 ± 4.4 mmHg, cardiac output (CO) 5.6 ± 0.9 L/min, and pulmonary vascular resistance (PVR) 668.6 ± 219.1 dynes.sec/cm^5^. The baseline 6MWD was 348.2 ± 35.6 meters. The use of PAH specific therapy improved mPAP by 7.54 mmHg (95% CI 10.2 to 4.9), CO by 1.77 L/min (95% CI 1.1 to 2.4), and PVR by 253 dynes.sec/cm^5^ (95% CI 291.4 to 214.6) (*n* = 135) and 6MWD by 61.8 meters (95% CI 47.5 to 76) (*n* = 122).* Conclusions*. The use of PAH specific therapy in PoPH results in significant improvement in both pulmonary hemodynamics and 6MWD.

## 1. Introduction

Portopulmonary hypertension (PoPH) refers to pulmonary arterial hypertension (PAH) associated with portal hypertension with or without cirrhosis [1]. The reported incidence ranges from 2 to 9% [2]. PoPH falls under group I of the WHO classification of pulmonary hypertension as it is histopathologically indistinguishable from idiopathic PAH [3]. It is the third most common cause of PAH after idiopathic PAH and PAH associated with connective tissue disease.

Pulmonary arterial hypertension specific therapy has been shown to result in significant improvement in pulmonary hemodynamics and exercise capacity in patients with idiopathic PAH and PAH associated with connective tissue disease [4]. However, little is known about the effectiveness of PAH specific therapy in patients with PoPH since such patients are usually excluded from randomized clinical trials (RCTs) of such therapy because of overall poor survival as well as concerns about adverse drug effects. The use of PAH specific therapy in patients with PoPH may, by causing systemic vasodilatation, potentially exacerbate the hyperdynamic circulatory state [5]. There is a concern that epoprostenol may worsen splenomegaly and cause hypersplenism [6, 7], and endothelin receptor antagonists (ERA) may worsen liver function [8].

The effect of PAH specific therapy on pulmonary hemodynamics is particularly important since PoPH may preclude a patient from undergoing liver transplantation (LT). Mild PoPH, that is mean pulmonary artery pressure (mPAP) < 35 mmHg, is associated with negligible perioperative risks, but moderate disease (mPAP 35–50 mmHg) is associated with a perioperative mortality of 50%, and a mPAP > 50 mmHg is universally fatal [2].

We performed a systematic review and meta-analysis of studies of PAH specific therapy in PoPH to determine the effect of such therapy on pulmonary hemodynamics and exercise capacity as measured by six-minute walk distance (6MWD).

## 2. Materials and Methods

### 2.1. Search Methods

We searched PubMed on September 23, 2013, using the terms “(Therapy/Broad (filter)) AND (portopulmonary hypertension).”

### 2.2. Inclusion Criteria

We included studies that met the following criteria: ≥5 patients, AND PoPH confirmed by right heart catheterization (RHC), AND follow-up RHC data, AND/OR baseline and follow-up 6MWD data available. PoPH was defined as (i) mPAP > 25 mmHg; (ii) pulmonary vascular resistance (PVR) > 240 dynes*·*sec/cm^5^; and (iii) pulmonary artery wedge pressure (PAWP) ≤ 15 mmHg or transpulmonary gradient (mPAP-PAWP) > 12 [1].

### 2.3. Study Selection and Data Extraction

Two authors independently evaluated the eligibility of all studies to determine whether they met all inclusion criteria. Disagreements between two authors were resolved with discussion. The main data extracted from the studies included the following: (1) first author; (2) year of publication; (3) country; (4) study design; (5) number of patients treated with PAH specific therapy; (6) age; (7) sex; (8) cause of portal hypertension; (9) baseline pulmonary hemodynamic data; (10) follow-up pulmonary hemodynamic data; (11) baseline 6MWD; (12) follow-up 6MWD; (13) PAH specific drugs used; (14) duration of therapy; (15) data on LT; (16) data on the use of beta-blockers; and (17) any adverse drug effects.

### 2.4. Data Synthesis and Analysis

We used the freeware Meta-Analyst 3.13 and DerSimonian-Laird continuous 1-arm random-effects model to perform the analysis. Pooled effects on pulmonary hemodynamic parameters and 6MWD were presented as weighted mean differences with corresponding 95% confidence intervals (CIs). Forest plots were created for each outcome. Statistical heterogeneity was assessed using the Cochrane *Q* statistic (with *P* values < 0.10 considered significant). We also calculated *I* ^2^ statistics to estimate the proportion of variation attributable to between-study heterogeneity. In the studies in which the standard deviation (SD) of the mean change of the parameters studied was not provided, we obtained SD from standard error, CI, *t* values, or *P* values for differences in means [9].

## 3. Results

**Figure 1:**
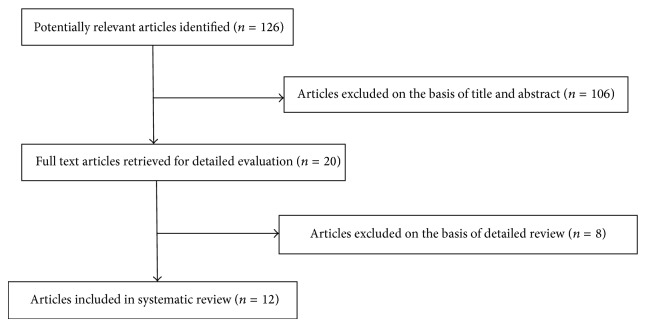
Flow diagram of literature search and selection of studies.

A total of 126 articles were retrieved. Twelve studies [10–21] met our inclusion criteria (Figure 1). Three were prospective in design and 9 retrospective. All but one were single arm studies. None was a RCT.

The mean ± SD age of the patients was 50.1 ± 3.6 years and 52.7% were males. The underlying cause of portal hypertension in the majority was alcoholic liver disease, chronic hepatitis B or C infection, or both (Table 1). The majority of patients belonged to Child class A. The baseline mPAP was 48.6 ± 4.4 mmHg, cardiac output (CO) 5.6 ± 0.9 L/min, and PVR 668.6 ± 219.1 dynes*·*sec/cm^5^. The baseline 6MWD was 348.2 ± 35.6 meters. The patients were treated with a variety of PAH specific therapy (Table 2). The duration of follow-up varied from as short as 1 month to as long as 4 years (Table 2).

**Figure 2:**
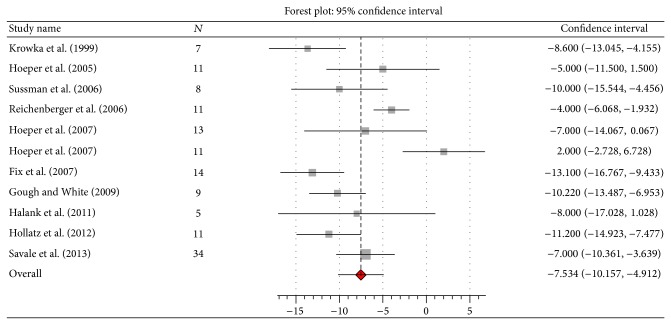
Effect of pulmonary arterial hypertension specific therapy on mean pulmonary artery pressure (mmHg).

**Figure 3:**
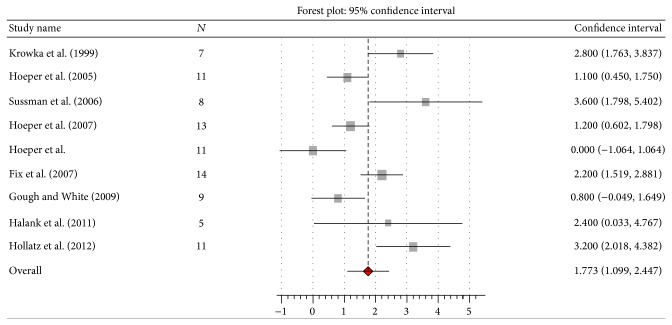
Effect of pulmonary arterial hypertension specific therapy on cardiac output (L/min).

**Figure 4:**
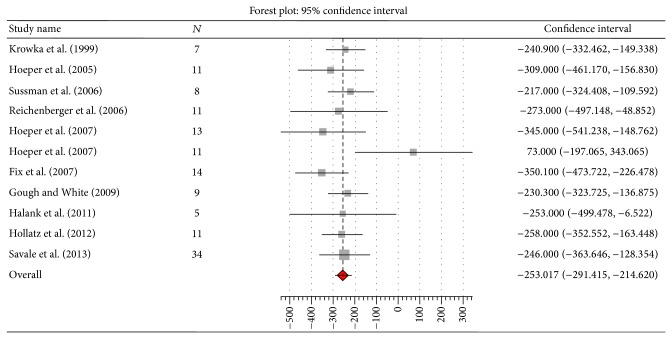
Effect of pulmonary arterial hypertension specific therapy on pulmonary vascular resistance (dynes*·*sec/cm^5^).

**Figure 5:**
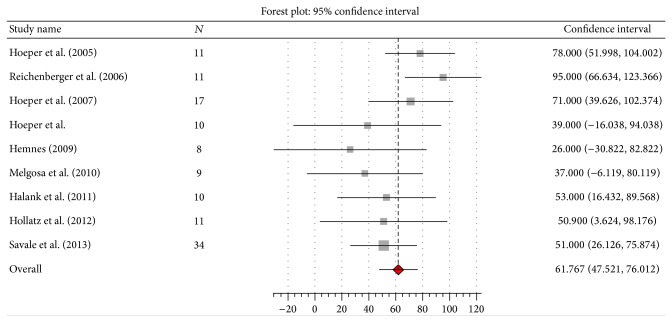
Effect of PAH specific therapy on 6-minute walk distance (meters).

The use of PAH specific therapy resulted in significant improvements in pulmonary hemodynamics (*n* = 135) and 6MWD (*n* = 122). The mPAP improved by 7.54 mmHg (95% CI 10.2 to 4.9; *I* ^2^ 77%, *Q* 43.2, *P* = 0.00), CO by 1.77 L/min (95% CI 1.1 to 2.4; *I* ^2^ 78%, *Q* 36.3, *P* = 0.00), PVR by 253 dynes*·*sec/cm^5^ (95% CI 291.4 to 214.6; *I* ^2^ 1%, *Q* 10.1, *P* = 0.43), and 6MWD by 61.8 meters (95% CI 47.5 to 76; *I* ^2^ 31%, *Q* 11.6, *P* = 0.17) (Figures 2, 3, 4, and 5). Seven studies [10, 13, 14, 16–18, 20] reported data on liver transplantation (Table 3). Only two studies reported data on the use of beta-blockers [12, 20]. No patients were on such therapy in one study [12] and it was discontinued in the other [20].

There were significant adverse drug effects in 3 (11%) of 28 patients treated with epoprostenol. One of them developed massive splenomegaly and progressive thrombocytopenia (65,000 to 6,000 over 18 months of epoprostenol) and died of sepsis following splenic embolization and subsequent splenectomy [10]. The drug was discontinued due to thrombocytopenia, hypotension, headache, and central venous line infections in the other two patients [14]. There was a significant elevation of liver transaminases (>3x the ULN) in 8 (11%) of 72 patients treated with ERAs (bosentan, *n* = 58; ambrisentan, *n* = 14) [15, 20]. The elevation resolved with dose reduction or drug discontinuation. Most of the patients belonged to Child class A cirrhosis and none to class C. There were no significant adverse drug effects in patients treated with PDE5I.

## 4. Discussion

This meta-analysis suggests that the use of PAH specific therapy results in significant improvement in pulmonary hemodynamics in patients with PoPH. The mean improvement in mPAP of 7.5 mmHg from a mean baseline mPAP of 48.6 mmHg, however, may not be clinically significant since a mPAP < 35 mmHg is required to safely proceed with LT [22]. Moreover, there was considerable heterogeneity among the studies. Nevertheless, cases of successful LT were reported in the studies that reported such data (Table 3). Interestingly, the most pronounced mean improvement in mPAP was also seen in these studies: Sussman et al. (−10 mmHg), Fix et al. (−13 mmHg), Gough et al. (−10 mmHg), and Hollatz (−11 mmHg) (Figure 2). This appears to be for the following two reasons: the mean baseline mPAP was lower in these studies compared to the others (Table 2), and patients were treated with the intention of bringing the mPAP down to <35 mmHg to allow LT to be performed safely, a goal which required the use of more than one PAH specific drug in a number of cases. It may be concluded that the use of a combination of PAH specific drugs may be required in some patients with PoPH to reduce the mPAP to the level acceptable for LT. However, no particular PAH specific drug or combination of drugs, dosing regimen, or duration of therapy can be suggested since a variety of drugs and dosing regimens were used and duration of therapy varied considerably among the studies.* Pulmonary hypertension may rarely persist following LT and warrant continuation of PAH specific therapy [9, 14, 20].*

This meta-analysis also suggests that the use of PAH specific therapy results in significant improvement in 6MWD. The improvement in 6MWD of 61.8 m is clinically significant, since the minimal clinically important difference in 6MWD in PAH is considered to be 41 m [23]. Although the possibility of a placebo effect cannot be excluded, the significant improvement in CO suggests the improvement in 6MWD is a result of it rather than a placebo effect. Nevertheless, the interpretation of these results is problematic because whether or not beta-blockers were prescribed or withdrawn for these patients was reported in only two studies [12, 20]. Beta-blockers are often used in patients with portal hypertension to reduce the risk of variceal bleeding. In patients with moderate to severe PoPH (mPAP > 35 mmHg) they are associated with significant worsening in pulmonary hemodynamics and exercise capacity. Withdrawal of beta-blocker therapy is associated with an increase in CO and exercise capacity [24].

Adverse drug effects were not uncommon. The rate of significant adverse effects requiring drug discontinuation in patients treated with epoprostenol was 11%. Although splenomegaly and hypersplenism were reported in only one of 14 patients in the series by Krowka et al. [10], a subsequent publication by the same group [6] reported its development in 4 (30%) of 13 patients; however, it is not clear if these were the same patients. Thrombocytopenia was not significant in the series by Fix et al. [14], whereas platelet counts were not reported in the series by Sussman et al. [13]. Nevertheless, splenomegaly with hypersplenism was recently reported in 5 (45%) of 11 patients with PoPH treated with epoprostenol and it was reversible on stopping the drug [7], a phenomenon not reported in the previous study [6] because the drug was not discontinued in any of the patients.

The rate of significant elevation of liver enzymes in patients treated ERAs was 11%. This rate is similar to that reported in those with other forms of PAH treated with ERAs [25–27]. Such elevation usually develops gradually, remains asymptomatic, and is generally reversible either spontaneously or after dose reduction or discontinuation. It is important to note, however, that none of the patients treated with ERAs in the studies included in this review belonged to Child class C cirrhosis. There were no significant adverse drug effects in patients treated with PDE5I.

Two studies that were excluded from this meta-analysis because their data could not be pooled are worth mentioning. In one study, the use of PAH specific therapy, mainly epoprostenol, in 16 patients with moderate to severe PoPH resulted in a significant improvement in mPAP to allow LT to be safely performed in 11 of them [28]. In another study, the use of ambrisentan in 11 patients with moderate to severe PoPH resulted in significant improvement in mPAP without adverse effect on liver function; however, LT had been performed in only one patient at the time of writing [29].

## 5. Conclusions

The use of PAH specific therapy in PoPH results in significant improvement in both pulmonary hemodynamics and 6MWD. The results of this meta-analysis should, however, be interpreted with caution, since the studies analyzed were uncontrolled case series of a small number of selected patients with moderate to severe PoPH, most of whom belonged to Child class A. A variety of PAH specific drugs were used, in combination in some cases. The duration of follow-up varied considerably among the studies. It is not surprising that there was considerable heterogeneity for mPAP and CO among the studies. Lastly, our search was only limited to PubMed. The use of PAH specific therapy in PoPH needs to be evaluated in RCTs in order to determine the most appropriate treatment regimen in terms of both efficacy and safety.

## Figures and Tables

**Table 1:** Underlying causes of portal hypertension in the included studies [10–21].

1st author and year	*N*	ALD	HBV or HCV	Cryptogenic	ALD + HCV	Miscellaneous
Krowka 1999	14	1	1	3	3	1 HCC + HCV, 1 sarcoidosis, 2 PBC, 1 Budd Chiari, 1 AIH
Hoeper 2005	11	7	1	0	2	1 biliary atresia
Reichenberger 2006	14	7	3			3 PBC, 1 Budd Chiari
Sussman 2006	8	4	1	2	1	
Fix 2007	35	7	9	3	10	1 HIV, 2 HCC, 2 PBC, 1 AIH
Hoeper 2007 (bosentan)	18	11	3	1	0	1 PVT, 2 AIH
Hoeper 2007 (iloprost)	13	6	2	0	0	1 biliary duct atresia, 4 AIH
Gough and White 2009	11	4	2	0	4	1 ALD + NASH
Hemnes 2009	13	1	8	0	0	1 PBC
Melgosa 2010	13	2	7	0	0	1 hemochromatosis, 2 PBC, 1 portal vein thrombosis
Halank 2011	13	8	1	3	0	1 PBC
Hollatz 2012	11	5	1	0	3	1 ALD + NASH + PBC, 1 biliary atresia
Savale 2013	34	20	4		3	6 PVT, 1 AIH

Total		83 (40%)	43 (20%)	12 (5%)	26 (12%)	41 (20%)

ALD: alcoholic liver disease; HBV: hepatitis B; HCV: hepatitis C; HCC: hepatocellular cancer; PBC: primary biliary cirrhosis; AIH: autoimmune hepatitis; HIV: human immunodeficiency virus; PVT: portal vein thrombosis; NASH: nonalcoholic steatohepatitis.

**Table 2:** Regimens of pulmonary arterial hypertension specific therapy used in the included studies [10–21].

1st author and year	*N*	Baseline mPAP in mm Hg Mean ± SD	PAH specific therapy	Dose Median (range) unless otherwise specified	Duration of therapy in months Total or median (range)
Krowka 1999	7	50 ± 13.4	Epoprostenol	11 (7–48) ng/kg/min	6 (3–30)
Hoeper 2005	11	53 ± 9	Bosentan	250 mg/day	12
Reichenberger 2006	12	55 ± 11	Sildenafil ±iloprost	150 mg/day 30 mcg/day (*n* = 5)	12
Sussman 2006	8	43	Epoprostenol	2–8 ng/kg/min	4.5 (2–15)
Fix 2007	14	47.9 ± 8.5	Epoprostenol ± another^*^	29 (6.5–50.5) ng/kg/min	15.4 (6.2–69.8)
Hoeper 2007	13	53 ± 8	Bosentan	250 mg/day	12
Hoeper 2007	11	50 ± 10	Iloprost	30 mcg/day	12
Gough and White 2009	9	47.6 ± 9.9	Sildenafil	150 (60–400) mg/day	5.6 (3.2–9.4)
Hemnes 2009	10	47.8 ± 12.1	Sildenafil	60–150 mg/day	12
Melgosa 2010	12	55 ± 10	Iloprost	30 mcg/day	12
Halank 2011	5	47 ± 6	Ambrisentan	5 or 10 mg/day	12
Hollatz 2012	11	44.4 ± 5.5	Sildenafil +/or SQ trepostinil	120 (60–150) mg/day 32 (19–53) ng/kg/min	7 (1–48)
Savale 2013	34	50 ± 10	Bosentan	250 mg/day	4–12

^*^Sildenafil (and occasionally bosentan, inhaled iloprost, or subcutaneous treprostinol) was added if the response to epoprostenol was considered by the treating physician to be inadequate, or if side effects greatly limited the ability to achieve an adequate infusion rate of epoprostenol.

**Table 3:** Data on liver transplantation [10, 13, 14, 16–18, 20].

1st author and year	*N* treated with PAH specific therapy	*N* eligible for LT	*N* who underwent LT successfully	*N* alive and awaiting LT	*N* died awaiting LT
Krowka 1999 [10]	7	4	2	2	
Sussman 2006 [13]	8	6	4		2
Fix 2007 [14]	14	5	2		3
Gough and White 2009 [16]	9	6	1	2	2
Hemnes 2009 [17]	10	3	1		1
Melgosa 2010 [18]	12	?	2		
Hollatz 2012 [20]	11	11	11		

*N*: number of patients; LT: liver transplantation.
